# Unique clinicopathological features of metaplastic breast carcinoma compared with invasive ductal carcinoma and poor prognostic indicators

**DOI:** 10.1186/1477-7819-11-129

**Published:** 2013-06-06

**Authors:** Yanni Song, Xiaolong Liu, Guoqiang Zhang, Hongtao Song, Yanlv Ren, Xiaoguang He, Yanbo Wang, Jinfeng Zhang, Youxue Zhang, Shanshan Sun, Xiaoshuan Liang, Qian Sun, Da Pang

**Affiliations:** 1 Department of Breast Surgery, The Third Affiliated Hospital of Harbin Medical University, 150 Haping Road, Harbin 150081, China; 2 Department of Pathology, The Third Affiliated Hospital of Harbin Medical University, 150 Haping Road, Harbin 150081, China; 3 Lovelace Health System Pathology, Lovelace Medical Center, 601 Dr Martin Luther King, Albuquerque, NM 87106, USA; 4 Department of Tumor Surgery, Heilongjiang Province Land Reclamation Headquarters General Hospital, 235 Hashuang Road, Harbin 150088, China; 5 Heilongjiang Institute for Cancer Research, 6 Baojian Road, Harbin 150040, China

**Keywords:** Breast cancer, Clinical outcomes, Metaplasia, Pathologic features, Prognosis

## Abstract

**Background:**

Metaplastic breast carcinoma is a rare aggressive malignant neoplasm. The purposes of this study are to review the pathologic features and clinical outcomes of metaplastic breast carcinoma compared to invasive ductal carcinoma and to evaluate the prognosis of metaplastic breast carcinoma.

**Methods:**

The cases of 55 patients with metaplastic breast carcinomapresenting between 1991 and 2006 were analyzed and compared to the cases of 767 age-matched patients with invasive ductal carcinoma from the same time period.

**Results:**

The group of patients with metaplastic breast carcinoma presented with a larger tumor size, lower lymph node involvement, higher percentage of triple-negative (estrogen receptor-, progesterone receptor- and human epidermal growth factor receptor-2-negative) cases, and Ki-67 over-expression compared with the group of patients with invasive ductal carcinoma and triple-negative invasive ductal carcinomas. Patients in the metaplastic breast carcinoma group tended to have more local (often chest wall) recurrences (*P* = 0.038) and distant (often lung) metastases (*P* = 0.001) than those in the invasive ductal carcinomas group. The prognosis of metaplastic breast carcinoma was poorer than that of invasive ductal carcinoma and triple-negative invasive ductal carcinomas; the 5-year overall survival rate was 54.5% in metaplastic breast carcinoma versus 85.1% in invasive ductal carcinoma, and 73.3% in triple-negative invasive ductal carcinomas (*P* <0.001). The 5-year disease-free survival rate was 45.5% in metaplastic breast carcinoma versus 71.2% in invasive ductal carcinoma, and 60.3% in triple-negative invasive ductal carcinomas (*P* <0.001). Multivariate analysis revealed tumor size larger than 5.0 cm, lymph node involvement and Ki-67≥14% were significantly related to 5-year overall survival (*P* = 0.010; *P* = 0.010; *P* = 0.035) and 5-year disease-free survival (*P* = 0.020; *P* = 0.018; *P* = 0.049).

**Conclusions:**

Metaplastic breast carcinoma shows a poorer prognosis than both invasive ductal carcinoma and triple-negative invasive ductal carcinomas. Tumor size larger than 5.0 cm, lymph node involvement and Ki-67 ≥14% indicate a poor prognosis in patients with metaplastic breast carcinoma.

## Background

Metaplastic breast carcinoma (MBC) is a rare malignancy characterized by various combinations of adenocarcinoma with mesenchymal and epithelial components. It exhibits a variety of histopathologic patterns and appears to be both epithelial and mesenchymal in origin. MBC accounts for less than 1% of all breast carcinomas [1]. Because it was not officially recognized as a distinct histopathologic subtype until 2000, knowledge about patient demographics, presentation, tumor characteristics, prognosis and treatment patterns is limited. The World Health Organization have classified MBC into pure epithelial-type and mixed epithelial and mesenchymal type [2]. The epithelial-type MBC is subclassified into squamous cell carcinoma (SCC), adenosquamous carcinoma (ASC) and adenocarcinoma with spindle cell differentiation (SPC); mixed type MBC is subclassified into carcinosarcoma (CS) and carcinoma with osseous and chondroid metaplasia (COC).

MBC differs from typical invasive ductal carcinoma (IDC) in several pathological and clinical aspects, and the prognosis and optimal treatment for MBC are largely not well studied. To date, only small series and case reports have attempted to delineate the factors that make MBC different from more common malignant breast cancer [3-5]. Recently, much attention has been paid to MBC because this neoplasm is usually characterized by a lack of estrogen receptor (ER), progesterone receptor (PR) and human epidermal growth factor receptor-2 (HER2) over-expression, which is called triple negativity [6]. Triple-negative breast cancer has been known to be resistant to conventional endocrine therapy for hormone receptor-positive breast cancer or targeted therapies such as trastuzumab for HER2 over-expressing breast cancer [7-9]. It has been reported that the risk of tumor recurrence of MBC is higher than in typical breast cancer. However, this remains controversial [10-12]. The purpose of this study is to clarify these controversies. To this end, we compared the clinicopathological characteristics, management and prognosis of 55 patients with MBC with those of 767 patients with IDC, including 131 triple-negative IDC (TN-IDC) patients, treated at the Third Affiliated Hospital of Harbin Medical University (3rd AHHMU) from 1991 to 2006.

We analyzed indicators that affect the prognosis of patients with MBC and that could potentially be used to optimize systemic treatment to prevent relapse in the future.

## Methods

### Patients’ characteristics

**Figure 1:**
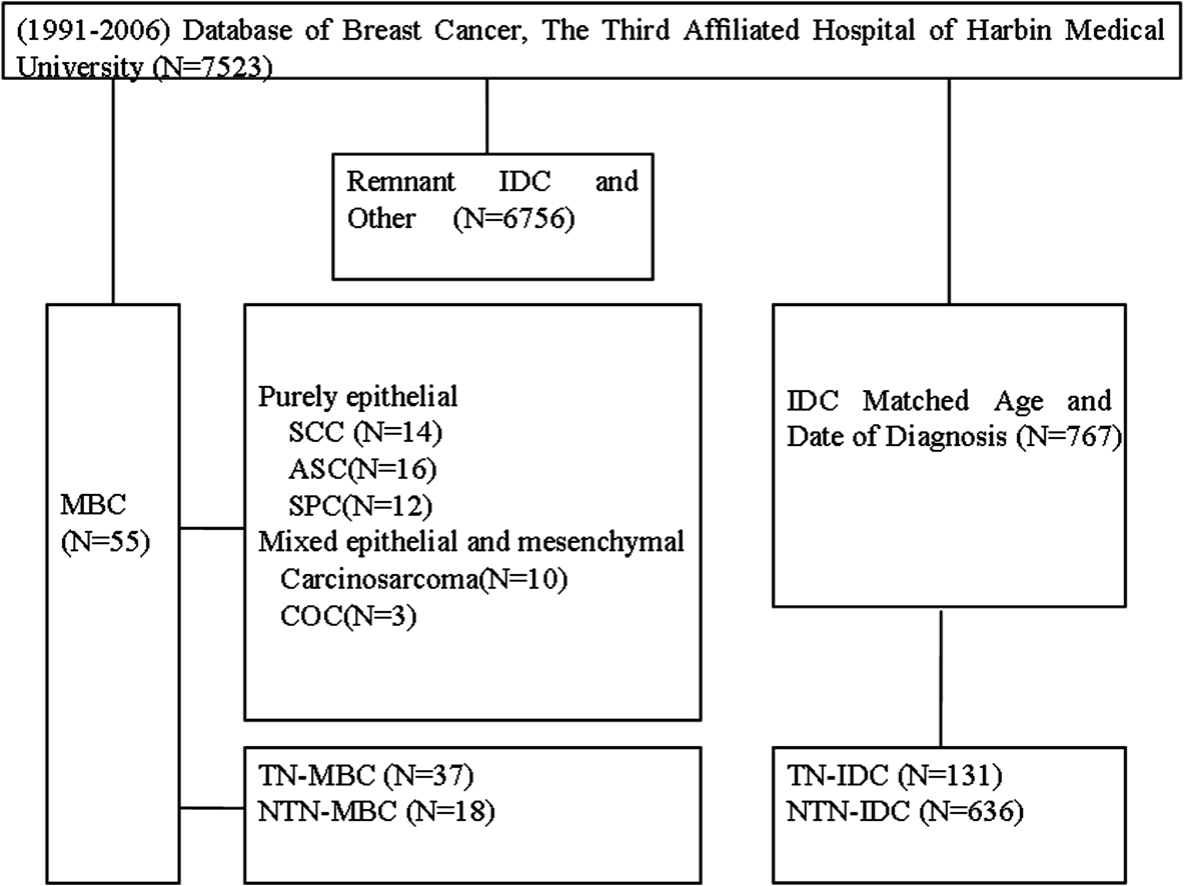
**Patient overview.** Patients treated between 1991 and 2006 were selected from the database of the Breast Cancer. ASC, adenosquamous carcinoma; COC, carcinoma with osseous and chondroid metaplasia; CS, carcinosarcoma; IDC, invasive ductal carcinoma; MBC, metaplastic breast carcinoma;NTN, not triple-negative; SCC, squamous cell carcinoma; SPC, adenocarcinoma with spindle cell differentiation; TN, triple-negative.

Patients were selected from the database of the Breast Cancer Center at the 3rd AHHMU between 1991 and 2006 (7,523 patients) (Figure 1). Only patients who had surgical pathological specimens of the primary tumor available for review were included. Fifty-five patients had been diagnosed with MBC and were included in this analysis; there were no patients with distant metastasis at the time of diagnosis. These available cases were compared with 767 IDC cases matched for age and period of diagnosis. The patients’ medical records were retrospectively reviewed to obtain demographic, clinicopathologic, treatment and prognostic information as well as the immunohistochemistry of biologic factors such as ER, PR, HER2, P53 and Ki-67. Patients with IDC who presented with a recurrent tumor, metastatic disease at presentation, male tumor, bilateral tumors, previous tumors in other sites, or those who had previously received neoadjuvant treatment were excluded. No patient received any therapy before surgery. All patients were followed until death or the study closing date (30 December 2011).

### Histopathology

At initial diagnosis, all tumor specimens were reviewed by two pathologists at the 3rd AHHMU. Tumor specimens were histologically examined, and cases were included in the re-review (55 MBC cases) if adenocarcinomatous elements were identified as admixed with SCC, spindle cell, chondroid or osseus tumor basing on hematoxylin and eosin staining and/or immunohistochemistry (IHC). Four-micron tissue sections, prepared from a formalin-fixed paraffin-embedded representative of the tumor samples, were used. The histological grade was determined according to the modified Bloom-Richardson classification. IHC staining for ER and PR (Zhongshan-Bio Co., Beijing, China) was performed using a conventional detection method and ER and PR status was evaluated based on the percentage of positively stained nuclei [13]. Positive staining for HER2 (Zhongshan-Bio Co.) was defined based on the percentage of tumor cells and the intensity of membrane staining. HER2 was scored as 0 to 3+ according to the method recommended for the DakoHercepTest. Tumors with scores of 3 or with a >2.2-fold increase in HER2 gene amplification as determined by fluorescence *in situ* hybridization were considered to be positive for HER2 over-expression [14]. Cells stained for Ki-67 and P53 were counted and expressed as a percentage. Low expression was considered as Ki-67<14% [15] and P53<25% [5]. All protocols were reviewed and approved by the Ethical Committee of Harbin Medical University in Harbin, China. Informed consent was obtained from all the patients.

For subgroup analysis, the definition of triple-negative breast cancer was as follows: negative ER and PR by IHC, and negative HER2 represented by an IHC score of 0 or 1+, or 2+ if not amplified by fluorescence *in situ* hybridization. The cases with an IHC score of 2+ for HER2 and no fluorescence *in situ* hybridization results were excluded from the triple-negative breast cancer group. In total, 131 of the control cases were classified as TN-IDC.

### Statistical methods

Overall survival (OS) was calculated from the date of surgery until death or the date patients were last known to be alive. The disease-free survival (DFS) was calculated from the date of surgery until relapse or the date patients were last known to be alive. The primary end points of this study were 5-year OS and 5-year DFS. In order to compare the clinicopathological characteristics between the two groups, we used the Student t-test and *χ*^2^ test. The 5-year OS and 5-year DFS rates were calculated using the Kaplan-Meier method and comparisons were made between MBC and control patients using the log-rank test. For multivariate analysis, Cox regression analysis was used. Statistical analyses were performed using SPSS 17.0 for Windows (Austin, TX, USA). A value of *P* <0.05 was considered statistically significant.

## Results

### Clinicopathological features

We retrieved 55 MBC cases from the 3rd AHHMU database, representing 0.73% of the 7,523 breast cancer cases. All patients were female, with a median age of 50 years (range, 24 to 71 years). The median tumor size was 5.0 cm (range, 1.5 to 20.0 cm). ASC was the most common histological subtype of MBC (N = 16), followed by SCC (N = 14), SPC (N = 12), CS (N = 10) and COC (N = 3). Clinicopathological features and treatment were analyzed for 55 patients with MBC and 767 patients with IDC (Table 1).

**Table 1:** Clinicopathological features of metaplastic breast carcinoma, invasive ductal carcinoma and triple-negative invasive ductal carcinoma

	**Metaplastic breast cancer (N = 55)**	**Invasive ductal carcinoma (N = 767)**		**TN-IDC (N = 131)**	
**Features**	**N (%)**	**N (%)**	***P***	**N (%)**	***P***
**Age**			0.594		0.264
≤50	27 (49.09)	405 (52.80)		76 (58.02)	
>50	28 (50.91)	362 (47.20)		55 (41.98)	
**Stage**			0.001		<0.001
I	4 (7.27)	107 (13.95)		21 (16.03)	
II	30 (54.55)	522 (68.06)		92 (70.23)	
III	16 (29.09)	87 (11.34)		8 (6.11)	
Unknown	5 (9.09)	51 (6.65)		10 (7.63)	
**Operation**			0.051		0.054
Breast-conserving surgery	4 (7.27)	134 (17.47)		25 (19.08)	
Modified radical mastectomy	51 (92.73)	633 (82.53)		106 (80.92)	
**Chemotherapy**			0.302		0.295
Yes	48 (87.28)	627 (81.74)		106 (80.91)	
No	7 (12.73)	140 (18.25)		25 (19.08)	
**Radiotherapy**			<0.001		<0.001
Yes	27 (49.09)	177 (23.08)		29 (22.14)	
No	28 (50.91)	590 (76.92)		102 (77.86)	
**Hormone therapy**			<0.001		
Yes	13 (23.64)	522 (68.06)		—	
No	42 (76.46)	245 (31.94)		—	
**Pathological tumor stage**			<0.001		<0.001
T1 (≤2 cm)	5 (9.09)	317 (41.33)		58 (44.27)	
T2 (2–5 cm)	22 (40.00)	332 (43.29)		55 (41.98)	
T3 (>5 cm)	22 (40.00)	39 (5.08)		5 (3.82)	
Tx	6 (10.91)	79 (10.30)		13 (9.92)	
**Pathological nodal stage**			0.001		0.001
N0	35 (63.64)	314 (41.20)		50 (38.17)	
N1-3	15 (27.27)	385 (49.93)		69 (52.67)	
Nx	5 (9.09)	68 (8.87)		12 (9.16)	
**Histological grade**			0.167		0.285
I or II	29 (52.73)	461 (60.10)		77 (58.78)	
III	20 (36.36)	227 (29.60)		41 (31.30)	
Unknown	6 (10.91)	79 (10.30)		13 (9.92)	
**Ki-67**^**a**^			<0.001		<0.001
≥14%	47 (87.27)	486 (63.36)		80 (61.07)	
<14%	8 (12.73)	281 (36.64)		51 (38.93)	
**P53**^**b**^			0.001		0.008
≥25%	28 (50.91)	221 (28.81)		40 (30.53)	
<25%	27 (49.09)	546 (71.19)		91 (69.47)	
**Estrogen receptor**			<0.001		
+	8 (14.55)	419 (54.63)		—	
−	47 (85.45)	348 (45.37)		—	
**Progesterone receptor**			<0.001		
+	10 (18.18)	492 (64.14)		—	
−	45 (81.82)	276 (35.98)		—	
**Human epidermal growth factor receptor-2**			0.005		
+	9 (16.36)	268 (34.94)		—	
−	46 (83.64)	499 (65.06)		—	
**TN-MBC**	37 (67.27)	131 (18.22)	<0.001	—	
**Not TN-MBC**	18 (32.73)	636 (81.78)		—	

^a^Median value for Ki-67 = 13.96, ^b^Median value for P53 = 24.85. TN-MBC, triple-negative metaplastic breast carcinoma; TN-IDC, triple-negative invasive ductal carcinoma.

The MBC group presented with a significantly larger tumor size than the IDC group (>T2, 80% versus 48%, *P* <0.001) and with less nodal metastasis (negative nodal status, 64% versus 41%, *P* = 0.001). More patients in the MBC group had stage III disease at diagnosis (29% versus 11%, *P* = 0.001). The MBC group had significantly more cases with no hormone receptors or HER2 over-expression/gene amplification compared with the IDC group (ER-, 85% versus 45%, *P* <0.001; PR-, 82% versus 36%, *P* <0.001; HER2-, 84% versus 65%, *P* = 0.005). There were significantly more triple-negative cases in the MBC group compared with the IDC group (67% versus 17%, *P* <0.001). Fewer patients received adjuvant hormonal therapy in the MBC group than in the IDC group (24% versus 68%, *P* <0.001). Over-expression of Ki-67 was more common in the MBC group compared with the IDC group (Ki-67 ≥14%, 87% versus 63%, *P* = 0.001).

The patients in the MBC group received more radiotherapy and less hormonal therapy than those in the IDC group (radiotherapy, 49% versus 23%, *P* <0.001; hormonal therapy, 24% versus 68%, *P* <0.001). There was no difference in the rates of chemotherapy and surgery between the two groups. Sentinel lymph node (SLN) biopsy in MBC was successful in eight of nine (89%) patients. Negative SLNs (without metastasis in SLNs) were found in seven of eight (87%) patients. In seven patients with negative SLNs, no positive non-SLN was found. SLN was not compared with IDC and TN-IDC due to small patient numbers.

Table 2 gives the different locations of the recurrent sites of MBC and IDC. Patients with local recurrence in the MBC group presented more chest wall recurrence than those in the IDC group (*P* = 0.038). Distant metastases were significantly more likely to be in the lung in patients with MBC but in the bone in patients with IDC (*P* = 0.001).

**Table 2:** The different locations of the recurrent sites of metaplastic breast carcinoma and invasive ductal carcinoma

**Recurrent sites**	**MBC (N = 33)**	**IDC (N = 222)**	***P***
Local recurrence	10	27	0.038
Chest wall	10 (100%)	18 (67%)	
Axillary fossa	0	9 (33%)	
Distant metastasis	23	193	0.001
Lung and pleura	14 (61%)	50 (26%)	
Liver	4 (17%)	15 (8%)	
Bone	4 (17%)	116 (60%)	
Brain and meninges	1 (4%)	12 (6%)	
Others	0	2	

### Univariate analysis of the 5-year overall survival and 5-year disease-free survival

**Figure 2:**
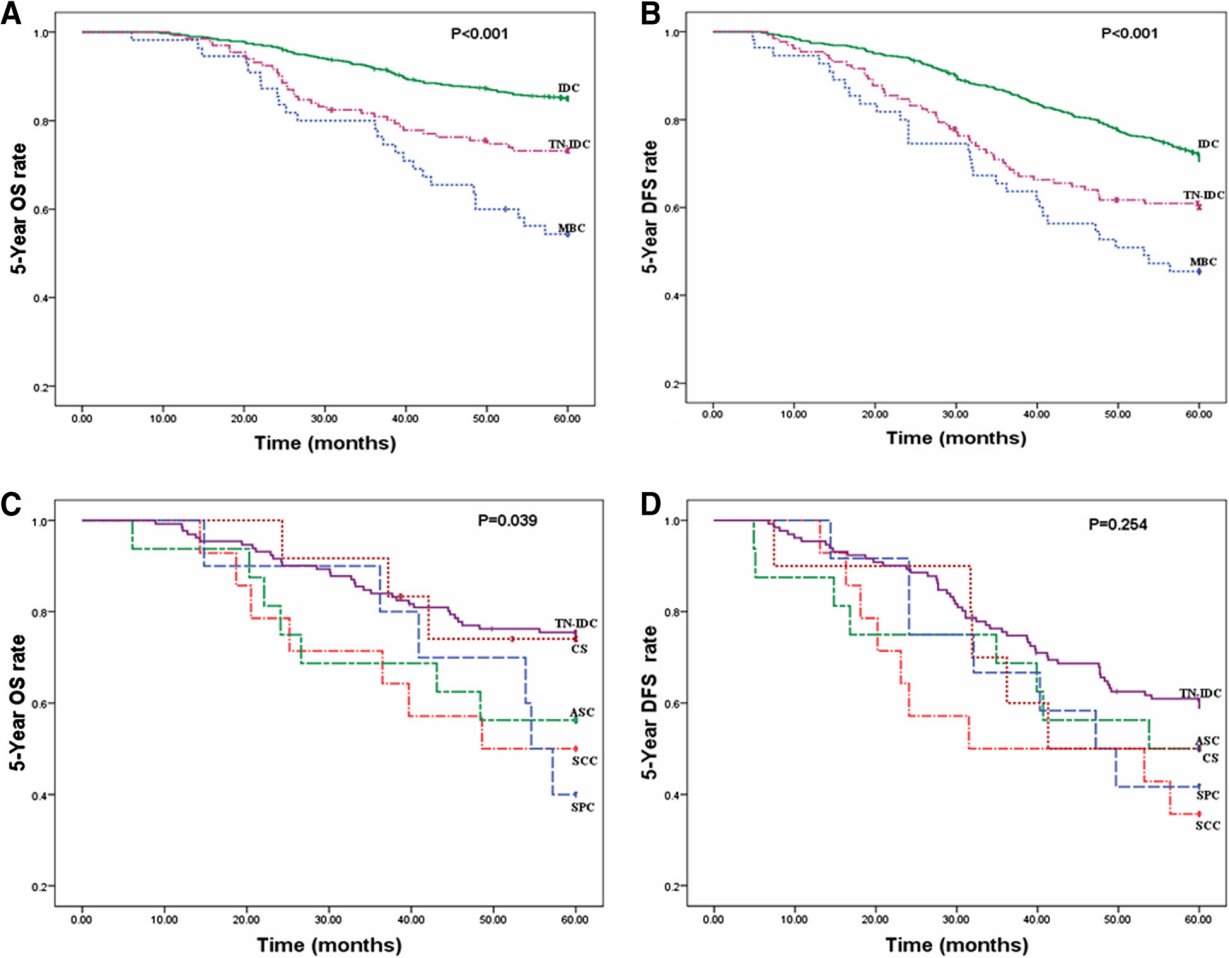
**Survival curves. (A)** Five-year OS curves based on MBC, IDC and TN-IDC. **(B)** Five-year DFS curves based on MBC, IDC and TN-IDC. **(C)** Five-year OS curves for SCC, ASC, SPC, CS and TN-IDC. **(D)** Five-year DFS curves for SCC, ASC, SPC, CS and TN-IDC.ASC, adenosquamous carcinoma; CS, carcinosarcoma; DFS, disease-free survival; IDC, Invasive ductal carcinoma; MBC, metaplastic breast carcinoma; OS, overall survival; SCC, squamous cell carcinoma; SPC, adenocarcinoma with spindle cell differentiation; TN-IDC, triple-negative invasive ductal carcinoma; TN-MBC, triple-negative metaplastic breast carcinoma.

Figure 2A, B presents the 5-year OS and 5-year DFS curves for MBC, IDC and TN-IDC. The prognosis was poorer for MBC than for IDC and TN-IDC, with a 5-year OS rate of 54.5% in MBC versus 85.1% in IDC and 73.3% in TN-IDC (*P* <0.001). The 5-year DFS rate was 45.5% in MBC versus 71.2%in IDC and 60.3% in TN-IDC (*P* <0.001).

Figure 2C, D presents the 5-year OS and 5-year DFS curves for TN-IDC and MBC subtypes. The prognosis of TN-IDC was better than any subtype of MBC, with a 5-year OS rate of 73.3% in TN-IDC versus 50.0% in SCC, 56.3% in ASC, 40.0% in SPC and 75.0% in CS (*P* = 0.039). With regards the 5-year OS, SPC had the worst prognosis of the MBC subtypesand CS had the best. Looking at the 5-year DFS, patients with SCC showed more recurrence (35.7%) than those with TN-IDC (59.5%), ASC (50.0%), SPC (41.7%) and CS (50.0%), although *P* values of 5-year DFS (*P* = 0.254) were not significant.

### Multivariate analysis of the 5-year overall survival and 5-year disease-free survival in metaplastic breast cancer

Multivariate analysis (Table 3) revealed that 5-year OS and 5-year DFS were significantly related to tumor size larger than 5.0 cm (5-year OS: hazard ratio (HR) 3.22, 95% CI 1.32 to 7.89, *P* = 0.010; 5-year DFS: HR 2.96, 95% CI 1.19 to 7.39, *P* = 0.020) and lymph node involvement (5-year OS: HR 3.17, 95% CI 1.31 to 7.67, *P* = 0.010; 5-year DFS: HR 1.59, 95% CI 1.08 to 2.35, *P* = 0.018). Furthermore, both 5-year OS and 5-year DFS were significantly related to Ki-67 ≥14% (5-year OS: HR 2.93, 95% CI 1.08 to 7.96, *P* = 0.035; 5-year DFS: HR 2.72, 95% CI 1.00 to 7.41, *P* = 0.049).

**Table 3:** Multivariate analysis of 5-year overall survival and 5-year disease-free survival in metaplastic breast cancer

		**5-year OS**			**5-year DFS**	
	**HR**	**95% CI**	***P***	**HR**	**95% CI**	***P***
Pathological tumor stage (>T3)	3.224	1.32 to 7.89	0.010	2.962	1.19 to 7.39	0.020
Pathological nodal stage (positive)	3.170	1.31 to 7.67	0.010	1.594	1.08 to 2.35	0.018
Histological grade (3)	0.945	0.24 to 3.70	0.935	1.066	0.28 to 4.07	0.925
P53 (≥25%)	3.274	0.40 to 26.72	0.268	3.413	0.43 to 27.38	0.248
Ki-67 (≥14%)	2.926	1.08 to 7.96	0.035	2.719	1.00 to 7.41	0.049
Hormone therapy (yes)	0.290	0.06 to 1.42	0.126	0.341	0.07 to 1.68	0.185
Subtypes of MBC (SPC)	1.388	0.54 to 3.59	0.499	—	—	—

MBC, metaplastic breast cancer;SPC, adenocarcinoma with spindle cell differentiation.

### Systemic therapy

Of the 55 patients with MBC, 48 received adjuvant chemotherapy. Table 4 details the adjuvant chemotherapy regimens administered to these patients along with the treatment outcomes. In 15 of these 48 patients, radiation therapy was administered subsequent to chemotherapy; in two of these patients, further disease progression occurred, with isolated recurrences to the chest wall (four months after primary therapy) and liver (six months after primary therapy). At last follow-up of the 55 patients with MBC, 33 had experienced disease relapse, including 10 patients with locoregional recurrence, and 23 had experienced distant metastasis. Of these 23 patients, 14 (60.9%) had metastases to the lungs. The 23 patients were treated with various chemotherapy regimens for metastatic disease, including anthracyclines, carboplatin, taxanes, capecitabine, vinorelbine and trastuzumab. Only five patients (21.7%) had a partial response to therapy, and five patients (21.7%) had disease stabilization.

**Table 4:** Adjuvant chemotherapy regimens and relapses in 48 patients with metaplastic breast cancer

**Chemotherapy regimen**	**Number**	**Patient outcome**
Cyclophosphamide, methotrexate and fluorouracil	7	Seven relapsed.
(Fluorouracil), doxorubicin/anthracyclines and cyclophosphamide	7	Two relapse-free at 45 and 57 months. One alive and well at 62 months after a local recurrence. Two progressed while receiving adjuvant therapy. Two relapsed.
(Fluorouracil), doxorubicin/anthracyclines and cyclophosphamide to taxane, paclitaxel/cisplatin/carboplatin	9	Five relapse-free at 30, 53, 66, 69 and 101 months respectively. Four relapsed.
Taxane/paclitaxel, anthracyclines and cyclophosphamide	18	Eight relapse-free at 55, 64, 75, 91, 108, 110, 123 and 156 months respectively. Ten relapsed.
Taxane, anthracyclines and cyclophosphamidein combination with cisplatin/capecitabine	4	Three alive and well at 17, 25 and 39 months after a local recurrence. One alive at 45 months before a local recurrence, alive at 20 months after distant metastasis.
Paclitaxel, anthracyclines and cyclophosphamidein combination with capecitabine/vinorelbine	3	Three alive at 24, 29 and 41 months before a local recurrence, alive at 15, 18 and 25 months after distant metastasis.

### Radiotherapy

Of the 55 patients with MBC, 27were treated with adjuvant radiation therapy. Five of these 27 patients had a locoregional recurrence. The 5-year OS rate of the 28 patients without adjuvant radiation therapy was 40% and the 5-year DFS rate was 60%, which were not different from those of the patients treated with radiation therapy. Fifteen patients received radiation therapy for recurrent disease,and four patients had more than one site irradiated. All patients experienced a partial response or symptomatic improvement but, in five patients, disease progression occurred within the irradiated site (two locoregional, one lung, one meningeal and bone) in an average of 4 months.

### Hormonal therapy

Of the 55 patients with MBC, 13were treated with tamoxifen as adjuvant treatment. Of these 13 patients, 8 (61.5%) are currently alive and relapse-free. Tamoxifen was used for two patients with metastases, one of whom had an ER-positive tumor, but neither of these patients responded to the treatment.

## Discussion

MBC is a rare disease that accounts for less than 1% of all mammary tumors [1]. In our study, the incidence rate of MBC was only 0.73%. Interestingly, we found that an increasing number of patients with MBC were reported each year. The World Health Organization only recognized MBC as a distinct pathological entity in 2000. The increased incidence we noted may represent an actual increase in the disease. Alternatively, it may be a result of improved awareness and recognition by pathologists [4,5], who, since the early immunohistochemical reports more than 10 years ago [16], have increasingly recognized and reported this distinct histologic type of breast cancer. However, because of its rarity, only relatively small series have been reported.

The prognosis of MBC had not been well delineated. Although some investigators have reported a better prognosis for MBC than IDC, others had reported that the prognosis for MBC was unfavorable compared to IDC [4,17,18]. We observed a dismal prognosis for patients with MBC when compared with 767 patients with IDC, including 131 patients with TN-IDC. Most previous studies have found that the tumor was large at the time of MBC diagnosis. The median tumor size in our study was 5.0 cm (range, 1.5 to 20.0 cm), larger than the size of an IDC (median 2.3 cm) or TN-IDC (median 2.1 cm). Pezzi*et al*. [4] reported that the larger sizes of MBC at clinical presentation appeared to result from a more rapid growth rate.

MBC presented with axillary nodal involvement less frequently than did IDC of the breast. Only 15 of the 55 patients with MBC (27%) had nodal involvement in our study. These data were consistent with previous reports that showed incidences of axillary nodal involvement at diagnosis of MBC between 6% and 28% [19-21]. MBC was usually associated with a lower incidence of axillary nodal involvement than that of an IDC with similar size. Patients with MBC had a median tumor size of 5.0 cm. Data from patients with an adenocarcinoma of the breast without metaplasia suggested that, for tumors ranging in size from 2.0 to 4.0 cm, the expected frequency of axillary node involvement was greater than 50% [12]. In our study, only nine of 40 patients with MBC (22%) with tumor sizes between 3.0 and 5.0 cm were axillary node-positive. SLN biopsy was adaptive in patients with MBC before axillary lymph node dissection. Despite MBC being less likely to present with positive axillary lymph nodes, the risk of developing metastatic disease was greater than in typical adenocarcinoma of the breast. It was believed that hematogenous spread was more common in MBC. The above data support the concept that MBC is an aggressive tumor with a high risk of recurrence following the primary site therapy.

In our study, there was a very low incidence of hormone receptor positivity in MBC compared to IDC. Hormonal therapy was rarely provided to patients with MBC, consistent with the low incidence of hormone receptor positivity in these patients. Rosen [22] noted that the lack of ER and PR might be due to the absence of a prominent glandular epithelial compartment in these tumors. Previous studies have found HER2 over-expression ranging from 4% to 17% [9,23]. In our study, nine of 35 patients with MBC (26%) had HER2 over-expression, consistent with previous studies. Triple-negative cases accounted for 67% of MBC, within the range of previous studies, where 64% to 96% of patients with MBC showed triple-negativity [5,9].

Notably, mastectomy was performed more often for patients with MBC. This was likely due to a larger tumor: tumor size was >5 cm in 40% of patients with MBC compared with only 5% of patients with IDC. As a consequence of the lower rate of breast conservation and less nodal involvement, postoperative radiation therapy was performed less often for patients with MBC; however, these patients underwent chemotherapy more often due to their negative hormone receptor status and larger tumor size. In our study, patients who underwent mastectomy had an unfavorable survival rate during the follow-up period.

Recently, significant progress had been made in the field of MBC biology, which could hypothetically explain its far more aggressive nature compared with other triple-negative breast cancers. Lien *et al*. [24] elucidated and validated that the epithelial-mesenchymal transition-related genes were differentially up-regulated in MBC compared with IDC, and Hennessy *et al*. [25] demonstrated that MBC was distinct from basal-like cancers. MBC showed a close relationship with basal-like cancers and a novel subgroup of receptor-negative breast cancers. The patients with the features of both MBC and basal-like cancers had enrichment for stem cell-like markers with an elevation of CD29/CD24 and CD44/CD24 ratios.

Further research on the mechanisms of carcinogenesis and the potential importance of new molecular markers (for example, P63, Zinc Finger E-box Binding Homeobox 1 (ZEB1), B-cell lymphoma 2 (Bcl-2)) are needed to develop better prognostic factors for this disease. If our data were confirmed, patients with MBC, particularly those with metastatic disease, would be appropriate candidates for clinical trials evaluating new combinations of the active chemotherapy agents for breast cancer. Considering the differences in the clinical and biologic behavior of these tumors compared with IDC and TN-IDC, clinical trials designed specifically for MBC would be very helpful to further characterize the biology and therapy of this disease. In this study, the number of MBC cases is too small to convincingly determine the prognostic factors that affect treatment outcomes of MBC compared with IDC, therefore more cases and longer follow-up may be necessary for this type of analysis.

## Conclusions

MBC had distinct clinicopathological features, which include a larger tumor size at presentation, higher Ki-67proliferation index, and a higher proportion of ER-negative and/or PR-negative tumors compared to IDC and TN-IDC. Our study demonstrated that MBC was an extremely aggressive disease and showed poorer prognosis compared with general IDC and TN-IDC. We found that better systemic treatment was required to prevent recurrence. Poor prognostic indicators for MBC include a tumor size larger than 5.0 cm, lymph node involvement and Ki-67 ≥14%.

## Abbreviations

ASC: Adenosquamous carcinoma; COC: Carcinoma with osseous and chondroid metaplasia; CS: Carcinosarcoma; DFS: Disease-free survival; ER: Estrogen receptor; HER2: Human epidermal growth factor receptor-2; HR: Hazard ratio; IDC: Invasive ductal carcinoma; IHC: Immunohistochemistry; MBC: Metaplastic breast carcinoma; OS: Overall survival; PR: Progesterone receptor; SCC: Squamous cell carcinoma; SLN: Sentinel lymph node; SPC: Adenocarcinoma with spindle cell differentiation; TN-IDC: Triple-negative invasive ductal carcinoma; TN-MBC: Triple-negative metaplastic breast carcinoma.

## Competing interests

The authors declare that they have no competing interests.

## Authors’ contributions

YS, XL, HS and DP conceived the study, carried out the analyses and wrote the manuscript. GZ and YR provided patients and samples, obtained follow-up data and helped to draft the manuscript. XH, YW, SS and JZ added experimental data, participated in the interpretation of the data and in writing the manuscript. XL, QS and YZ performed the statistical analysis. All authors read and approved the final manuscript.
